# Dimethyl Sulfate as Methylation Agent and Solvent in Highly Regioselective Synthesis of Methyl Salicylate Using Sodium Bicarbonate as a Base

**DOI:** 10.1021/acsomega.4c10962

**Published:** 2025-03-29

**Authors:** Milton de Souza Freitas, David Lee Nelson, João Victor G. de Sousa, Alexandre P. Wentz, Dayane B. Tada, Rafaela C. Queiroz, Carolina R. Hurtado, Erenilda F. de Macedo, Katia Conceição, Gabriela R. Hurtado, Fernando L. P. Pessoa, Yan Valdez S Rodrigues, Gabriel de P. Bueno, Giuliano C. Clososki, Sandro L. Barbosa

**Affiliations:** † Department of Pharmacy, Universidade Federal dos Vales do Jequitinhonha e Mucuri-UFVJM, Campus JK, Rodovia MGT 367 - Km 583, n° 5.000, Alto da Jacuba, CEP 39100-000 Diamantina, MG, Brazil; ‡ Nanomaterials and Nanotoxicology Laboratory, Institute of Science and Technology, Federal University of São Paulo (UNIFESP), R. Talim, 330, Vila Nair, CEP 12231-280 São José dos Campos, SP, Brazil; § Federal Institute of São Paulo (IFSP), Rod. Pres. Dutra, km 145, Jardim Diamante, CEP 12223-201 São José dos Campos, SP, Brazil; ∥ Peptide Biochemistry Laboratory, Institute of Science and Technology, Federal University of São Paulo (Unifesp), R. Talim, 330, Vila Nair, CEP 12231-280 São José dos Campos, SP, Brazil; ⊥ Institute of Science and Technology, São Paulo State University (UNESP), Rod. Pres. Dutra, km 137,8, Eugênio de Melo, CEP 12247-004 São José dos Campos, SP, Brazil; # Institute of Advanced Sea Studies (IEAMAr), São Paulo State University (UNESP), Rod. Pres. Dutra, km 137,8, Eugênio de Melo, CEP 12247-004 São José dos Campos, SP, Brazil; ∇ Centro Universitário SENAI-CIMATEC, Av. Orlando Gomes, 1845, Piatã, Salvador 41650-010, Brazil; ○ Research Center for Natural and Synthetic Products, Faculty of Pharmaceutical Sciences of Ribeirão Preto, University of São Paulo (USP), Av. do Café, CEP, 14040-903 Ribeirão Preto, SP, Brazil

## Abstract

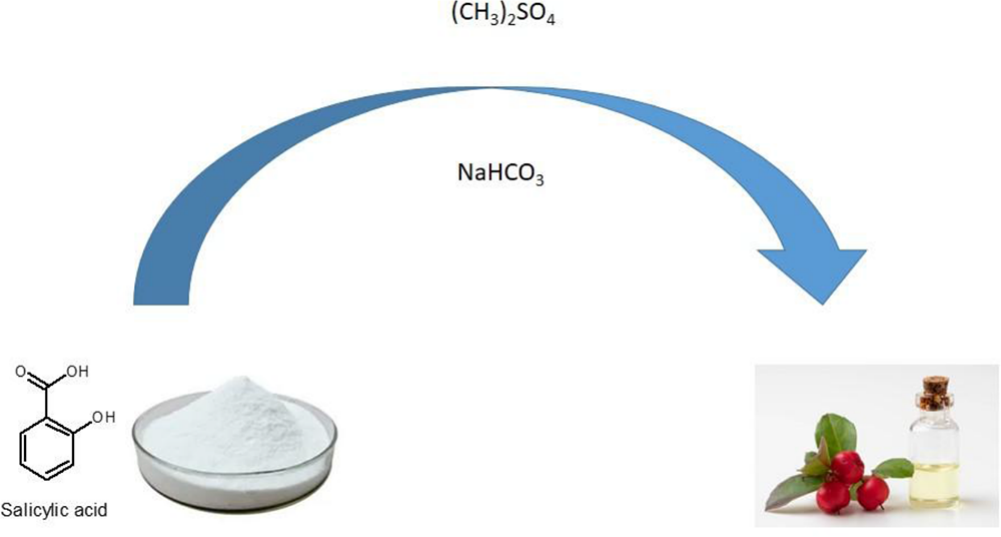

Methyl salicylate (MS), the principal constituent of wintergreen oil, was obtained from salicylic acid (SA) by regioselective methylation of the carboxyl group. A new procedure involved exclusive capture of carboxylic hydrogen (−CO_2_H) through the use of the selective base, NaHCO_3_, and methylation via an S_N_2 mechanism employing the previously formed carboxylate as a nucleophile and the dimethyl sulfate [DMS] as the electrophilic reagent or substrate in a solvent-free reaction process. SA and NaHCO_3_ were added, followed by DMS after 30 min, and the reaction mixture was stirred at 90 °C for 90 min. The reaction was accompanied by thin-layer chromatography and gas chromatography. The conversion rate via GC was 100%, and the MS yield was 96%. The DMS used in excess was transformed into MeOH and H_2_SO_4_ when the mixture was washed with water. The MeOH was stored, and H_2_SO_4_ was transformed in Na_2_SO_4_ by neutralization with NaOH. The simplicity of the procedure, ready availability of MS, short reaction times, excellent yields, and mild reaction conditions are other advantages of this protocol. MS is being biologically tested as an antibacterial and antitumor agent. The focus of the study is on the search for drugs with greater selectivity for tumor cells so as to reduce adverse effects on normal cells because MS is rarely reported in the literature for this application. Cytotoxicities of 50 and 64% for cultured *S. aureus* and metastatic melanoma cells, respectively, were observed for a concentration of 0.6 mg/mL of the MS produced, whereas no cytotoxicity against nontumor cells was observed at this concentration. This is considered to be the optimum concentration.

## 1 Introduction

*Gaultheria procumbens* L. (Wintergreen) is a small ericaceous plant cultivated for use in the landscape industry, and it is the source of the essential oil of wintergreen (WO).^1,2^ WO is obtained commercially by steam-distillation; however, the most commonly used form of WO is synthetic. Wintergreen oil is commonly used as a flavoring agent, but its leaves were historically used by North American natives for the treatment of aches and pains because of their analgesic activity.^2^ In fact, methyl salicylate (MS), the most common salicylate in commercial wintergreen preparations, is routinely used in topical ointments for the treatment of inflammation.^2^

With regard to the biological properties of WO, it has been demonstrated that some plants produce salicylic acid (SA) as a response to the infection by tobacco mosaic virus (TMV).^3^ Neighboring plants also develop resistance to TMV because the infected plants convert the SA to MS, which, being more volatile, is released into the air and signals the neighboring plants to increase their resistance to TMV.

Oloyede^4^ demonstrated the antimicrobial activity of the essential oil from *Laportea aestuans* (Gaud). The principal constituents in this oil were MS (54.50%), fenchol (10.59%), 1,2-cyclohexanedione dioxime (9.40%), 1,4-octadiene (8.86%) and linalool (3.26%). Activity against *Escherichia coli*, *Staphylococcus aureus* (*S. aureus*), *Bacillus subtilis*, *Pseudomonas aeruginosa*, *Klebsiella pneumoniae*, *Salmonella typhi*, *Candida albicans*, *Rhizopus stolon*, *Aspergillus niger*, and *Penicillium nonatum* was observed with the oil, and part of this activity might be due to the presence of MS.

A review of the literature regarding the toxicity of MS when ingested orally arrived at an allowable daily intake of 11 mg/kg/d.^5^ MS can be ingested from various sources, including chewing gum, baked goods, syrups, candy, beverages, ice cream, and tobacco products. Vlachojannis et al.^6^ explored the antimicrobial activity of Listerine, which is a popular mouthwash that contains MS as one of its components. This mouthwash is now composed of a 27% ethanol solution containing thymol, menthol, eucalyptol, and MS. The antimicrobial activities of individual Listerine components and mixtures have been studied, including the activities against *Enterococcus faecalis*, *Streptococcus mutans*, *Eikenella corrodens*, and **Candida albicans*.* The minimum inhibitory concentrations (MIC) and the minimum bactericidal/fungicidal concentrations (MBC/MFC) were used in the studies. No combination of two phenols at the concentrations contained in Listerine were observed to exert additive or synergistic effects. The same degree of activity was observed against the yeast with thymol as with Listerine. A combination of three of the phenols also exhibited the same activity against the bacteria as Listerine. Similar studies have been performed by other authors.^7−10^

Essien et al.^11^ studied the antimicrobial activity of volatile constituents from fresh fruits of *Alchornea cordifolia* and *Canthium subcordatum*. *A. cordifolia* oil contained 25.3% MS, whereas the oil from *Canthium subcordatum* contained only 4.5%. A potent in vitro antibacterial activity against *Staphylococcus aureus* (MIC = 78 μg/mL) and marginal antifungal activity against **Aspergillus niger** (MIC = 156 μg/mL) were observed for the essential oil from *A. cordifolia*. Antibacterial activities against *Bacillus cereus* and *S. aureus* (MIC = 156 μg/mL) and antifungal activity against **A. niger** (MIC = 39 μg/mL) were observed for the essential oil from *C. subcordatum*. However, no appreciable cytotoxic effects on human breast carcinoma cells (Hs 578T) and human prostate carcinoma cells (PC-3) were observed for either essential oil.

With regard to the method of synthesis utilized, esters can be produced by a large number of processes that include acid-catalyzed esterification of carboxylic acids with alcohol,^12^ the use of coupling agents such as *N*,*N*′-dicyclohexylcarbodiimide and reactive derivatives such as acid chlorides or anhydrides.^13^ These latter reactions avoid the production of water. Another pathway for producing esters involves the nucleophilic attack of the carboxylate anion on an electrophile. Baeyer–Villiger oxidation and oxidative esterifications can also be employed for the synthesis of esters.

Many other examples of ester synthesis can be mentione.^14−25^ Liu et al.^26^ observed that red mud, which is an alkaline residue containing various metal oxides, is effective for producing biodiesel from oils. Mazzocchia et al.^27^ obtained fatty acid methyl esters from triglycerides using heterogeneous catalysis under microwave radiation.

**Scheme 1:**
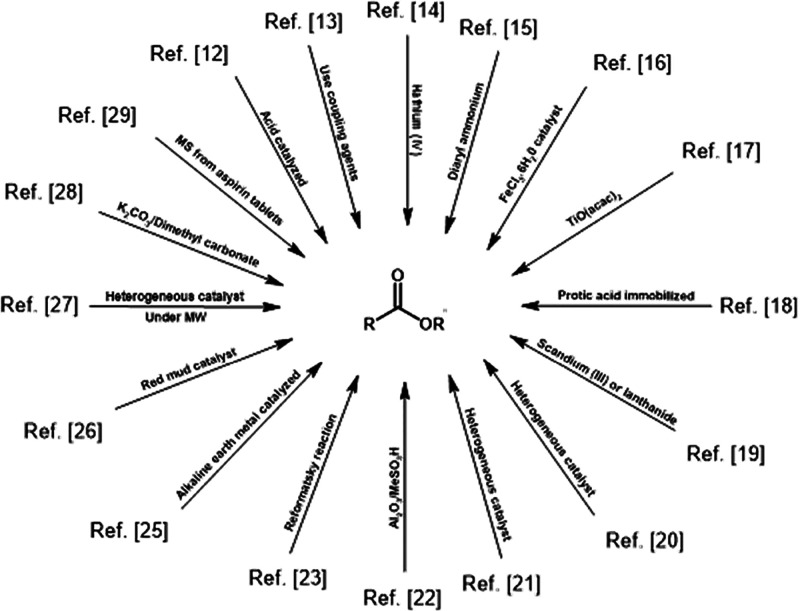
Methods for Selective Methylation of Carboxylic Acids or Their Salts

Le et al. achieved the methylation of carboxylic acids using dimethyl carbonate as the source of the methyl group in a process promoted by K_2_CO_3_ as a base and tetrabutylammonium chloride as the phase transfer catalyst.^28^ Hanna Jr. et al. related the preparation of MS from commercial aspirin tablets.^29^ However, among all these examples, very little has been reported regarding the use of DMS for promoting selective esterification. In one example, potassium acetate was esterified in the absence of solvent under phase transfer conditions using catalytic quantities of tetraalkylammonium salts.^30^ The general reaction Scheme 1 outlines the relevant established methods for selective methylation, highlighting their connection to this work and supported by references.^12−23,25−29^

Recently, we reported that MS was obtained from acetylsalicylic acid (ASA) in excellent yield (94%), using 20% w/w of the catalyst SiO_2_–SO_3_H, with a surface area of 115 m^2^/g, a pore volume of 0.38 cm^3^g^–1^ and 1.32 mmol H^+^/g, in a tandem transesterification-esterification process performed in a microwave reactor.^29^ We developed a procedure where the inorganic salt KH_2_PO_4_ participated as a regioselective base for the preparation of MS from SA using DMS in excess at 90 °C. A 96.23% conversion to MS and 3.77% conversion to methyl 2-methoxybenzoate were obtained.^31^

In the present work, sodium bicarbonate was studied for the conversion of SA into salicylate anion and the salicylate ion into MS in a process that involves nucleophilic aliphatic substitution. Sodium bicarbonate is more readily available and the price is much lower than that of KH_2_PO_4_. DMS was used as the esterification reagent (electrophilic reagent) or methylation agent, as well as the aprotic solvent for the reaction. Pure methyl 2-methoxybenzoate-free MS was synthesized in this laboratory as a demonstration of the utility of the synthetic process utilizing this reagent. The absence of methyl 2-methoxybenzoate facilitates the workup process and purification of the product. The cytotoxic potentials of the synthetic MS against metastatic melanoma cells, a bacterial culture of *S. aureus*, and nontumoral cells of fibroblasts were evaluated. The method of synthesis utilized can easily be scaled up for the production of MS for further studies without negative environmental effects.

## 2 Experimental Section

### 2.1 Raw Materials and Chemicals

All the reagents (analytical grade), including commercial MS, MeOH, and SA (SA standard), NaHCO_3_ and DMS were supplied by Vetec, São Paulo, Brazil and were used without further purification. Dulbecco’s Modified Eagle Medium (DMEM) (Gibco) was prepared in deionized water, buffered with sodium bicarbonate (Synth, Brazil) and supplemented with fetal bovine serum (FBS) (Vitrocell Embriolife). Streptomycin, ampicillin, 3-(4,5-dimethylthiazol-2-yl)-2,5-diphenyltetrazolium bromide (MTT), and dimethyl sulfoxide were purchased from Sigma-Aldrich (St. Louis, MO, USA). Mueller Hinton Broth Medium (MHB) (Kasvi) was prepared in deionized water.

### 2.2 Instrumentation

MS content and conversion rate were determined with a GC/MS-QP 2010/AOC 5000 AUTO INJECTOR/Shimadzu Gas Chromatograph/Mass Spectrometer equipped with a 30 m Agilent J&W GC DB-5 MS column. Direct insertion spectra were measured at 70 eV. Quantitative analyses were performed on a Shimadzu GC-2010 gas chromatograph equipped with a flame ionization detector.^32^^1^H- and ^13^C NMR spectra were recorded on Bruker *Avance* 400 Spectrometers as has been previously described.^32^ All the reactions were monitored by TLC using Silica Gel 60 F 254 on aluminum. The chromatograms were visualized by UV light or by using the ethanolic vanillin developing agent.^31^ The Purification of the products was achieved by flash column chromatography using a 9/1 mixture of hexane/ethyl acetate as the eluent.^32^

### 2.3 Typical Procedures

All experimental procedures were performed in a laboratory fume hood. The procedure utilized for the methylation process was based on various trials to determine the optimum conditions for this reaction.

### 2.4 Methylation of SA in DMS Using NaHCO_3_ as a Selective Base

To a 150 mL round-bottom flask coupled to a reflux condenser, 2.7624 g (20 mmol) of SA, and 1.6801 g (20 mmol) of NaHCO_3_ were added. The reaction mixture was heated on the heating mantle at 90 °C for 30 min, and DMS (2.5226 g, 1.92 mL, 40 mmol) was added with the help of a glass syringe. The mixture was magnetically stirred for another 90 min at a temperature of 90 °C. The reaction process was monitored by thin layer chromatography throughout this period. The mixture was cooled to room temperature, transferred to a separatory funnel, and 20.0 mL of deionized water and 10.0 mL CH_2_Cl_2_ were added. An aqueous phase containing MeOH and H_2_SO_4_^33^ was transferred to a beaker, and NaOH was added to pH 7. Na_2_SO_4_ (0.1790 g) was formed, recrystallized from water, dried in a muffle furnace (600 °C for 2h) and used as a desiccant. The organic solution obtained was removed and transferred to another extraction funnel and partitioned between 10 mL of CH_2_Cl_2_ and 20 mL of saturated NaHCO_3_; the organic phase was dried with Na_2_SO_4_, filtered, and evaporated under reduced pressure. The resulting residue was subjected to GC/MS analysis, which demonstrated the absence of unreacted SA. The residue was then purified by flash column chromatography on silica using hexane:ethyl acetate (9:1) as the mobile phase to yield MS (2.9203 g, 96%) as a colorless oil. This procedure was performed in triplicate, and the yields were repeatable. Due care should be exercised in the use of DMS because it is suspected to be a human carcinogen^34,35^ and has been shown to cause cancer in laboratory animals.^36−38^

### 2.5 Biological Activity

#### 2.5.1 Cytotoxicity Test

Mouse fibroblast L929 cells^39^ were obtained from the collection of the Laboratorio de Nanomateriais e Lanotoxicologia, where they were stored at −80 °C. They were cultured in Dulbecco’s Modified Eagle’s Medium (DMEM) supplemented with 10% (v/v) FBS, sodium bicarbonate (2 g/L), streptomycin (0.1 g/L), and ampicillin (0.025 g/L). Cells were incubated at 37 °C in a humidified atmosphere containing 5% CO_2_. L929 cells were seeded into 96-well polypropylene plates (10^4^ cells/well) and incubated for 24 h. The culture medium was removed and replaced with a fresh culture medium containing the various samples. The cells were treated with 0.025–3.6 mg/mL of the synthesized MS. After treatment, the cells were incubated for 24 h. The cells were washed with 100 μL of fresh PBS. The cell viability was evaluated by incubating the cells with 100 μL of an MTT (3-(4,5-dimethylthiazol-2-yl)-2,5-diphenyltetrazolium bromide) solution (0.5 mg/mL) for 3 h. The MTT solution was replaced by DMSO to dissolve the formazan crystals. The final absorbance of formazan was measured in a microplate reader (Synergy H1-Biotek) at 540 nm. The absorbances of cells incubated without samples were considered to represent 100% of viability. The cell viability data were analyzed by one-way ANOVA and Tukey’s multiple range method (*p* < 0.05) to determine statistical differences between different groups of samples.

#### 2.5.2 Antitumoral Activity

Murine melanoma B16F10-Nex2 cells^40^ were obtained from the collection of the Laboratory of Nanomaterials and Lanotoxicology, where they were stored at −80 °C. They were cultured in RPMI-1640 medium supplemented with 10% (v/v) FBS, sodium bicarbonate (2 g/L), streptomycin (0.1 g/L), and ampicillin (0.025 g/L). The cells were incubated at 37 °C in a humidified atmosphere containing 5% CO_2_. B16F10-Nex2 cells were seeded into 96-well polypropylene plates (10^4^ cells/well) and incubated for 24 h, following the same protocol described for the cytotoxicity test.

#### 2.5.3 Antibacterial Test

The antimicrobial activity was evaluated by using a modified NCCLS broth microdilution method.^41^ Antimicrobial activity was monitored using a liquid growth inhibition assay against *S. aureus* (ATCC 6538), which were obtained from the collection of the Laboratory of Nanomaterials and Lanotoxicology, where they were stored at −80 °C. The preinoculum of the strain was prepared in MHB (Mueller Hinton Broth Medium) for approximately 12 h at 37 °C. The inoculum was standardized as 10^6^ cells/mL by measuring the absorbance at 630 nm and plated into 96-well polypropylene plates. Bacterial cells were treated with 0.025–3.6 mg/mL of synthesized MS. After treatment, the cells were incubated for 24 h. The inhibition of bacterial growth was evaluated by measuring the absorbance at 600 nm after 24 h (Synergy H1-Biotek). The *S. aureus* grown in MHB in the absence of any samples was used as a negative control.

## 3 Results and Discussion

### 3.1 Synthesis of MS Using DMS as Methylation Agent

**Scheme 2:**
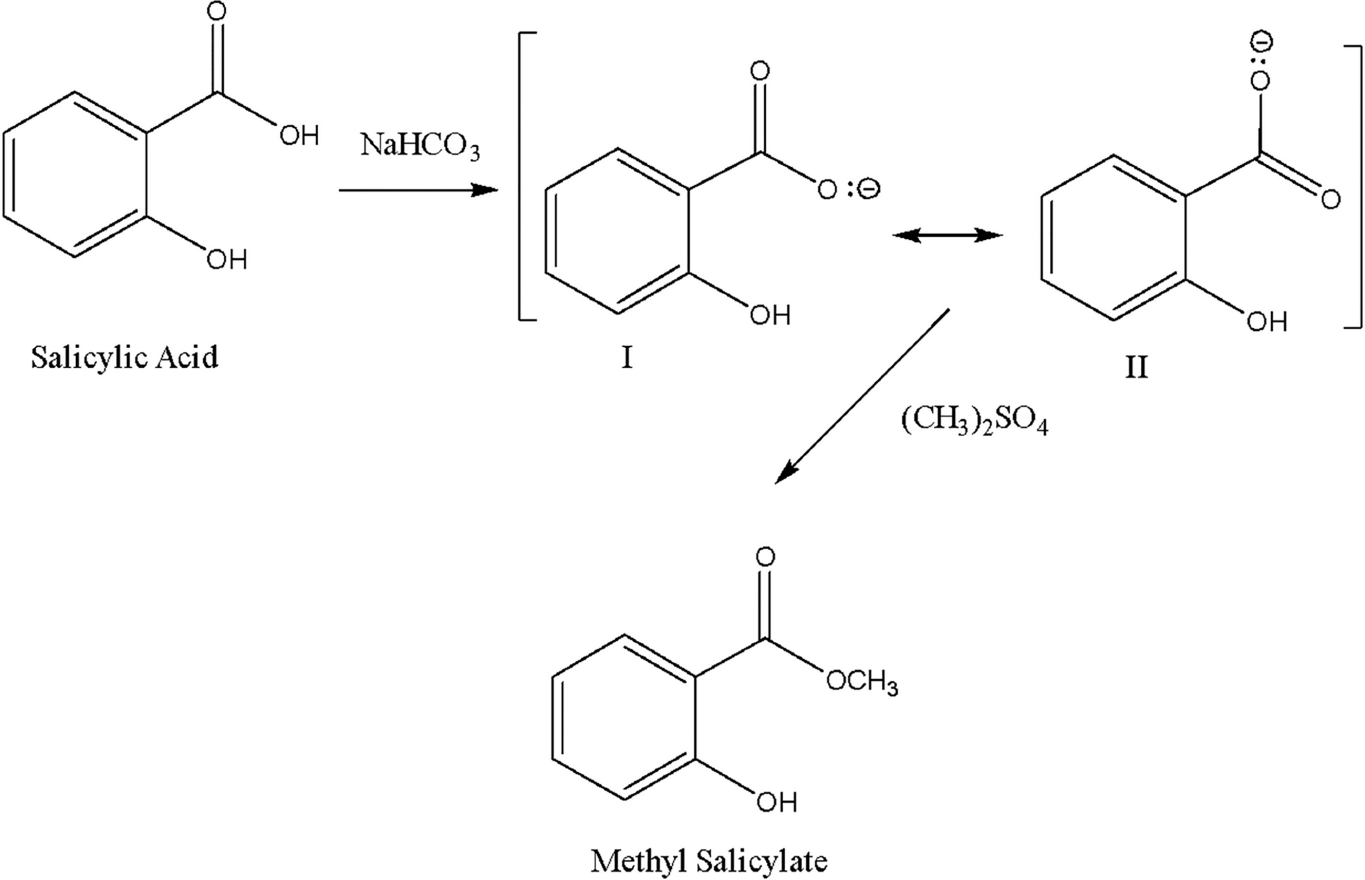
Synthesis MS from SA Using DMS as Methylation Agent by S_N_2 Mechanism

In the present study, we also explored the use of (CH_3_)_2_SO_4_ as a methylating reagent (as an electrophile) in an esterification reaction with the objective of obtaining a 100% conversion of SA to MS. We also investigated the use of the salicylate ion prepared “in situ” from salicylic acid in the synthesis of MS. In this new and innovative reaction process (Scheme 2), the selective reactivity of a weakly basic reagent, NaHCO_3_, was explored. The reaction occurs in the absence of an organic solvent other than the excess DMS, which is an important factor in avoiding solvent waste, as was mentioned by Sheldon.^42^

The carboxylate ion is resonance stabilized to a very significant extent, as the negative charge on oxygen is delocalized extensively between two electronegative oxygens. This resonance makes the carboxylate ion very stable, so the hydrogen from the carboxylic acid group is more acidic than that of phenols, which are also stabilized by resonance with the aromatic ring. Thus, carboxylic acids can release protons more easily than alcohols or phenols. In the absence of water, the basic nature of the bicarbonate ion is accentuated.^43^

NaHCO_3_ (sodium hydrogen carbonate) was chosen because it has a weakly basic nature. The phenolic hydroxyl group of MS does not react with NaHCO_3_, so it does not dissolve in aqueous NaHCO_3_. Carbonic acid is a stronger acid than most phenols, and, consequently, the equilibrium for their reaction with HCO_3_^–^ lies far to the left, eq 1.
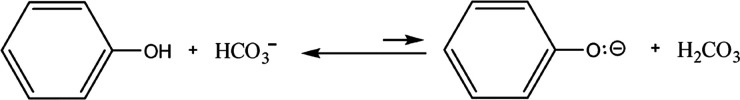
1

**Figure 1:**
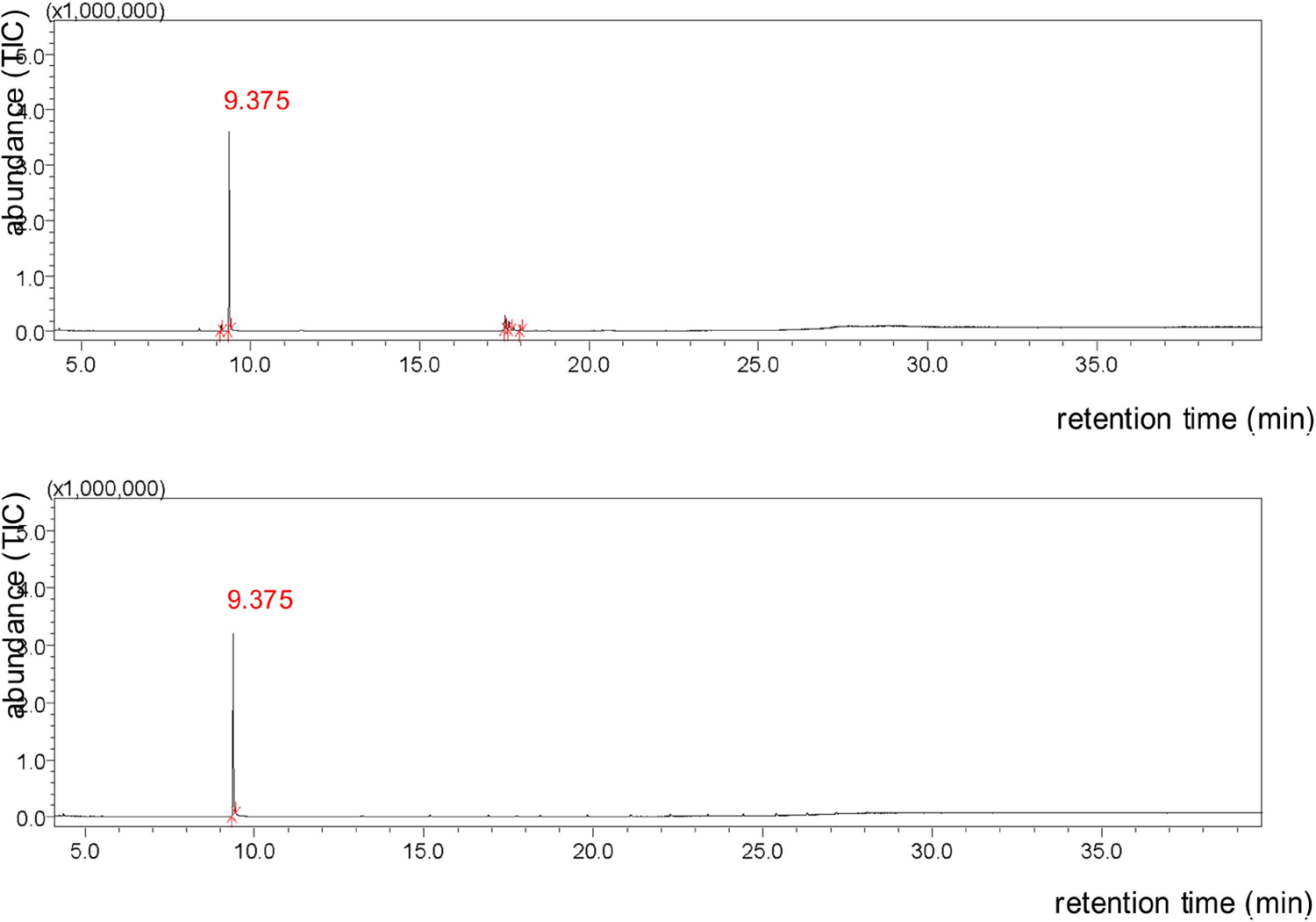
Chromatograms of the crude methylation product containing MS, SA and traces of other contaminants (upper) and the purified product (lower).

NaHCO_3_ is formed by a combination of a strong base and weak acid, converting it to a basic salt. A regioselective process involving SA occurred in which only carboxylate anions are formed as sources of oxygen nucleophiles in this process. We believe that the excess DMS served as a methylation agent and as a solvent for the reaction because the use of an excess led to the total consumption of the starting reagent, SA, and accelerated the reaction process. The progress of the reaction was monitored by thin layer chromatography (TLC) in hexane:ethyl acetate (9:1 v/v) as the eluent and also by gas chromatography, where we could confirm the total consumption of the substrate (SA). After completion of the methylation reaction, gas chromatography was used to measure the quantities of SA consumed and MS formed (Figure 1).

The reaction time (9.375 min) for total consumption of SA was 2 h. DMS is suspected to be a human carcinogen^33,42^ and can cause cancer in laboratory animals.^35−37^ The excess DMS is usually destroyed using NaOH, Na_2_CO_3_, and NH_4_OH.^43^ In this study, DMS was transformed into Na_2_SO_4_ by treatment with aqueous NaOH. DMS was dissolved in water with methylation of the water to form methyl hydrogen sulfate and free sulfuric acid as the result of the hydrolysis,^32^ (eqs 2 and 3).
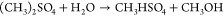
2
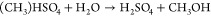
3

**Scheme 3:**
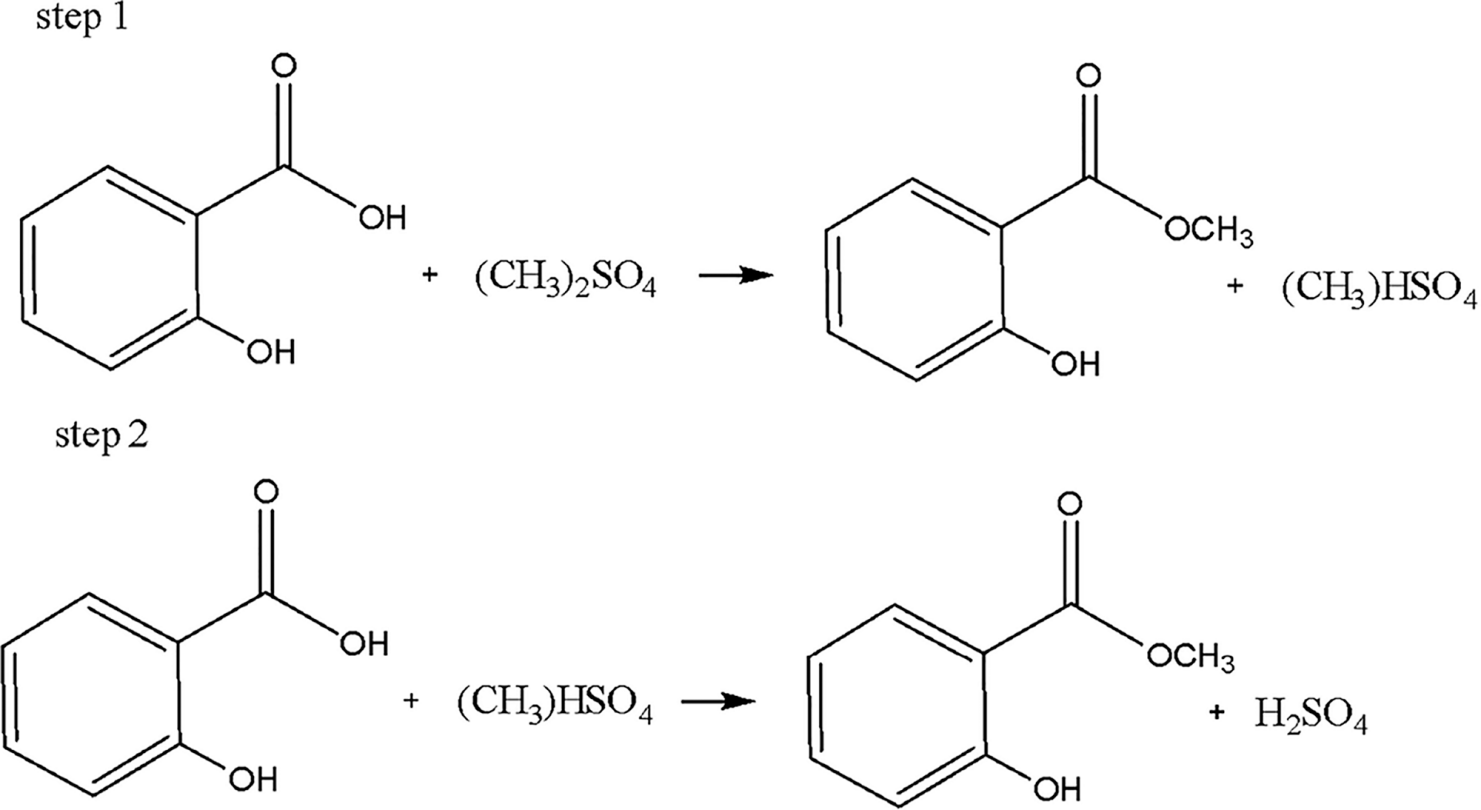
Methylation of SA with DMS Without the Addition of NaHCO_3_

The final reaction mixture containing Na_2_SO_4_ is not mutagenic or cytotoxic. The Na_2_SO_4_ is an excellent desiccant (eq 4). Thus, waste is minimized.
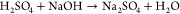
4When DMS (4.0 mmol) and SA (1.0 mmol) are mixed without the addition of NaHCO_3_ and heated to 90 °C, methyl hydrogen sulfate, (CH_3_)HSO_4_, and MS are slowly formed (step 1, Scheme 3). A 20% yield of MS was obtained after reacting for 48 h under these conditions (Scheme 3). The DMS was used in excess because it is an aprotic solvent for the reaction. When DMS (1.0 mmol or 2.0 mmol) and SA (1.0 mmol) are mixed without the addition of NaHCO_3_ and heated to 90 °C, no (CH3)HSO4 or MS is formed. When DMS (1.0 mmol or 2.0 mmol) and SA (1.0 mmol) are mixed with NaHCO3 (1.0 mmol) and heated to 90 °C, MS is formed in 45 and 70% yield, respectively. DMS only reacts via an S_N_2 mechanism because the methyl cation would be very unstable and the methyl group is not sterically hindered.

### 3.2 Study of the ^1^H NMR of MS

**Figure 2:**
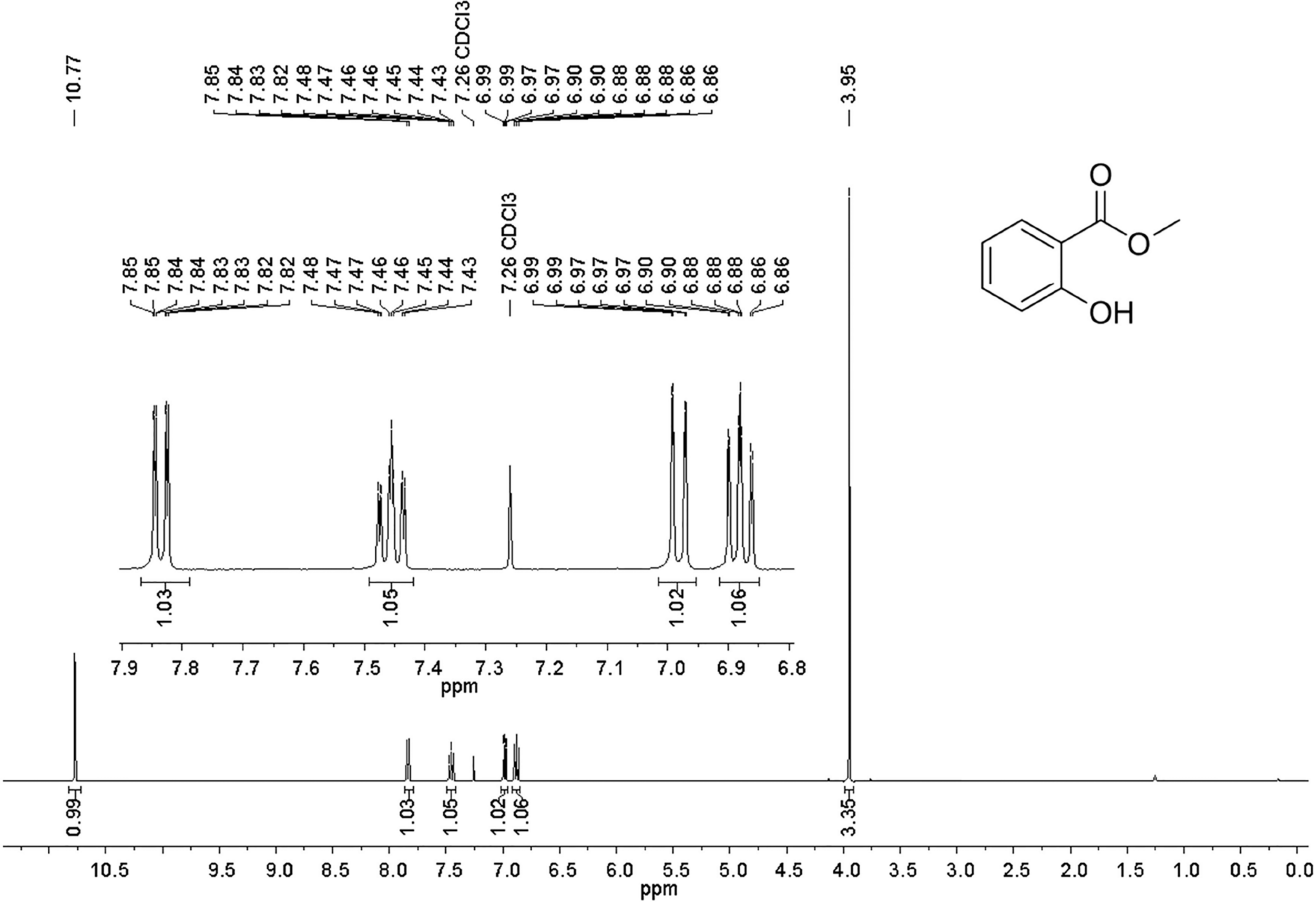
^1^H NMR (400 MHz, CDCl_3_) of MS.

The chemical shifts are quoted in ppm on the diagram of the ^1^H NMR spectrum of MS (Figure 2).

These data are in accordance with those of the literature.^44^

### 3.3 Study of the Biological Activity

**Figure 3:**
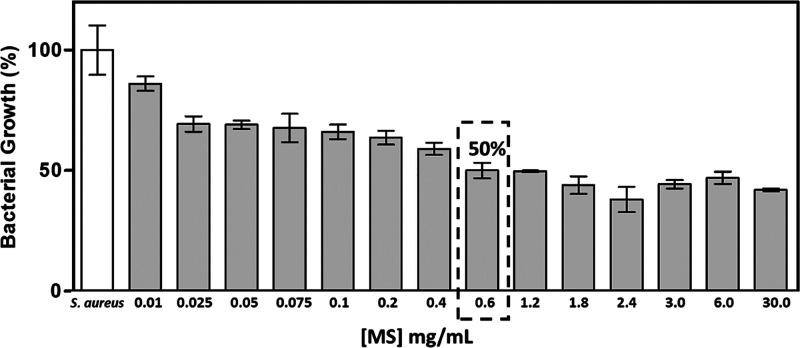
Antimicrobial activity of synthesized MS against *S. aureus*.

All of the biological assays were performed using the synthesized MS. A gradual decrease in bacterial growth was observed when *S. aureus* cells were exposed to increasing concentrations of MS, with the maximum antimicrobial activity (50% reduction in *S. aureus* growth) occurring at a concentration of 0.6 mg/mL of MS (Figure 3). No significant difference in antimicrobial activity was observed at concentrations greater than this optimum concentration.

Methyl salicylate (MS) is present in essential oils, particularly those of different *Gaultheria* species, along with other compounds that can contribute to antimicrobial activity.^45^ Ganiyat^4^ reported that an inhibitory activity was observed for the essential oil from *Laportea aestuans*, containing 54.50% MS, when it was employed against *S. aureus* at a concentration of 200 mg/mL, which corresponds to an MS concentration of 100 mg/mL. In the present study, synthesized and purified MS was used according to the described method, and a 50% inhibition of *S. aureus* growth was observed with a concentration of only 0.6 mg/mL, which demonstrated an antimicrobial activity greater than that observed with the essential oil from *L. aestuans*.

**Figure 4:**
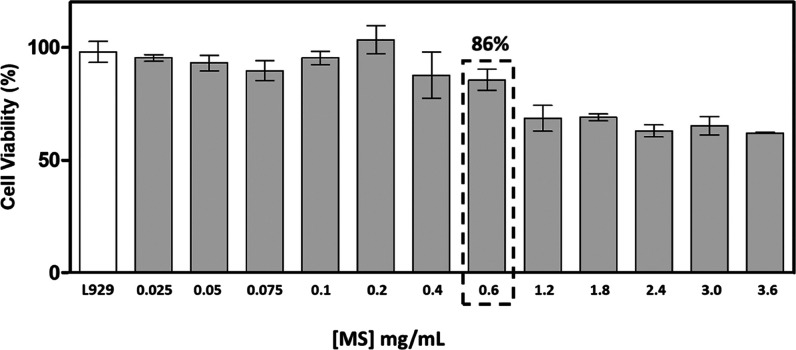
Cytotoxicity of MS in L929 cells.

After observing this antimicrobial effect of MS, the cytotoxic effect of different concentrations of MS in L929 fibroblast cells (nontumor) was studied. No cytotoxic effect was observed at concentrations of 0.025–0.6 mg/mL of MS, and, despite observing a decrease in cell viability, no cytotoxicity at concentrations greater than 0.6 mg/mL (Figure 4) was observed.

**Figure 5:**
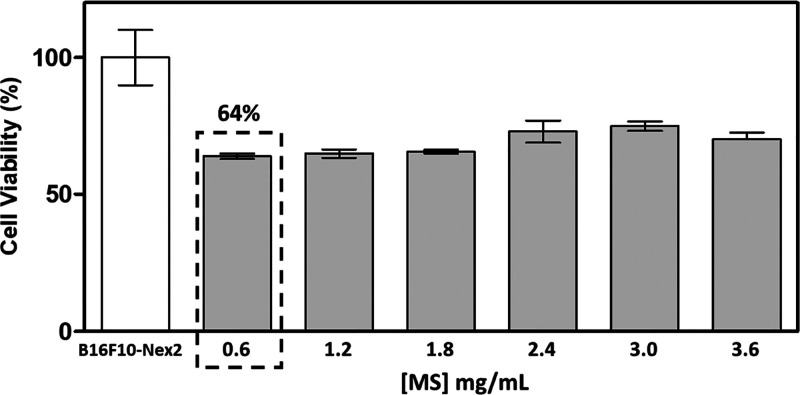
Cytotoxicity of MS in B16F10-Nex2 cells.

According to the established value of 70% cell viability as the minimum threshold for considering a material to be noncytotoxic (ISO 10-993-5),^46^ we concluded that the cytotoxicity of the pure MS obtained in this study was dose-dependent. Once the noncytotoxic concentration for fibroblast cells and the antimicrobial activity against *S. aureus* were defined, the cytotoxicity of MS in metastatic melanoma cells at the optimal concentration of 0.6 mg/mL was determined. A cell viability of 64% compared to the control without the presence of MS (Figure 5) was observed. At higher concentrations, no significant difference was observed in the reduction of cell viability compared to the optimal concentration.

Antitumor activity using Wintergreen essential oil, which is almost entirely composed of MS, is rarely reported in the literature. A low carcinogenic activity has been observed in rat neuroblastoma;^47^ however, Banerjee^48^ observed significant cytotoxicity in breast cancer cells (MDA-MB). These findings suggest that the antitumoral activity can be observed under specific conditions for different types of cells. In the present study, cytotoxicity was observed in metastatic melanoma cells at a concentration that is noncytotoxic for healthy cells, a finding that has not yet been described in the literature and warrants further studies for a better understanding of the mechanism of cell death.

The highest selectivity for tumor cells, reducing adverse effects on normal cells, is always the object of research. Thus, our study focused on using a dose that was noncytotoxic for healthy cells, but could have a cytotoxic effect against melanoma cells. The antiproliferative effect on cancer cells is mediated by a variety of pathways, where antitumor molecules must act by activating tumor-suppressor genes, negatively regulating cell cycle proteins, or promoting caspase-dependent apoptosis.^49−51^ The observation that a tumor cell exhibits genetic modifications that lead to disordered growth, which is different from healthy cells, is important. Thus, the cytotoxic mechanism depends, among other factors, on proteins and lipids present in the membranes, which allow for varying degrees of drug permeability so that drugs can potentially be cytotoxic to tumor cells but not to healthy cells. Therefore, regulatory effects on cell signaling pathways and genetic alterations have been widely studied as potential targets for new cytotoxic and antitumor strategies.^52^ For the specific case of MS, further studies are needed to understand the mechanism of cell death.

## 4 Conclusions

The use of DMS as a methylating agent demonstrated that the bimolecular nucleophilic substitution on an sp^3^ carbon occurred with total consumption of SA, demonstrated by GC, and involved the salicylate anion as the nucleophilic species and DMS as the substrate or electrophilic carbon source. DMS was also demonstrated to be an excellent aprotic solvent for the reaction, so it was used in excess. This innovative process involved the transformation of the DMS used in excess into a neutral and environmentally friendly reagent, Na_2_SO_4_. The reaction conditions utilized a heating mantle to supply the necessary energy, and the yield obtained was close to 100%. The concentration of 0.6 mg/mL of MS was observed to be cytotoxic for tumor cells. Lower cell viability was observed in cultured metastatic melanoma cells; and no cytotoxicity for nontumor fibroblast cells was observed at the same concentration. The synthetic MS obtained in this work has a potential for antimicrobial and possibly antitumoral applications in the absence of cytotoxicity for nontumor cells. More studies regarding these activities are desirable. The process is considered to be clean because the DMS (highly toxic) used during the reactions can be transformed into Na_2_SO_4_ (nontoxic) and reutilized as a desiccant. The MeOH was also recovered during the workup. The simplicity of the protocol leads us to believe that the process could easily be scaled up.

## Data Availability

The paper entitled “**Dimethyl sulfate as methylation agent and solvent in a highly regioselective synthesis of methyl salicylate using sodium bicarbonate as a base**” is submitted for your kind considerations. The research data policy and data availability are not shared with anyone before publication. All data generated or analyzed during this study are included in this published article and its Supporting Information files.

## Supporting Information Available

List of chemicals, NMR spectra, interpretation of ^1^H NMR spectrum and characterization data of MS compound, interpretation of 1H NMR spectrum, NMR spectra, interpretation of ^13^C NMR spectrum and characterization data of MS compound, interpretation of 13C NMR spectrum, and GC chromatogram of MS compound (PDF)

## Notes

This paper does not have any ethical statement, and this work does not involve any conflict of interest.
