# Supplementary material for: Dimethyl Sulfate as Methylation Agent and Solvent in Highly Regioselective Synthesis of Methyl Salicylate Using Sodium Bicarbonate as a Base
Source: ACS Omega. 2025 Mar 29;10(13):13260–8. doi: 10.1021/acsomega.4c10962 (PMC11983181; doi:10.1021/acsomega.4c10962)
Supplement: Supplementary file 1 — ao4c10962_si_001.pdf [file ao4c10962_si_001.pdf]

Dimethyl sulfate as methylation agent and solvent in highly regioselective synthesis of methyl salicylate using sodium bicarbonate as a base

Milton de Souza Freitas [1], David Lee Nelson [1], João Victor G. de Sousa [1], Alexandre P. Wentz [1], Dayane B. Tada [2], Rafaela C. Queiroz [2,3], Carolina R. Hurtado [2,3], Erenilda F. de Macedo [2], Katia Conceição [4], Gabriela R. Hurtado [5,6], Fernando L. P. Pessoa [7], Yan Valdez S. Rodrigues [7], Gabriel de P. Bueno [8], Giuliano C. Clososki [8], Sandro L. Barbosa [1*]

[1]Department of Pharmacy, Universidade Federal dos Vales do Jequitinhonha e Mucuri-UFVJM, Campus JK, Rodovia MGT 367 - Km 583, nº 5.000, Alto da Jacuba, CEP 39100-000, Diamantina/MG, Brazil. e-mail: freitas.milton@hotmail.com (M.d.S.F.); dleenelson@gmail.com (D.L.N.); sousa.joao@ufvjm.edu.br (J.V.S.); wentzap@hotmail.com (A.P.W.); sandro.barbosa@ufvjm.edu (S.L.B.)

[2]Nanomaterials and Nanotoxicology Laboratory, Institute of Science and Technology, Federal University of São Paulo (UNIFESP), R. Talim, 330, Vila Nair, CEP 12231-280, São José dos Campos/SP, Brazil. e-mail: d.tada@unifesp.br (D.B.T.); rafaelacamposqueiroz003@gmail.com (R.C.Q.); carolina.hurtado@ifsp.edu.br (C.R.H.); erenildafm@gmail.com (E.F.M.)

[3]Federal Institute of São Paulo (IFSP), Rod. Pres. Dutra, km 145, Jardim Diamante, CEP 12223-201, São José dos Campos/SP, Brazil. e-mail: carolina.hurtado@ifsp.edu.br (C.R.H.); rafaelacamposqueiroz003@gmail.com (R.C.Q.)

[4]Peptide Biochemistry Laboratory, Institute of Science and Technology, Federal University of São Paulo (Unifesp), R. Talim, 330, Vila Nair, CEP 12231-280, São José dos Campos/SP, Brazil. e-mail: katia.conceicao@unifesp.br (K.C.)

[5]Institute of Science and Technology, São Paulo State University (UNESP), Rod. Pres. Dutra, km 137,8, Eugênio de Melo, CEP 12247-004, São José dos Campos/SP, Brazil. e-mail: gabriela.hurtado@ict.unesp.br (G.R.H.)

[6]Institute of Advanced Sea Studies (IEAMAr), São Paulo State University (UNESP), Rod. Pres. Dutra, km 137,8, Eugênio de Melo, CEP 12247-004, São José dos Campos/SP, Brazil. e-mail: gabriela.hurtado@ict.unesp.br (G.R.H.)

[7]Centro Universitário SENAI-CIMATEC, Av. Orlando Gomes, 1845, Piatã, Salvador 41650-010, Brazil; e-mail: fernando.pessoa@fieb.org.br (F.L.P.P.); yanvaldez@gmail.com (Y.V.S.R.)

[8]Research Center for Natural and Synthetic Products, Faculty of Pharmaceutical Sciences of Ribeirão Preto, University of São Paulo (USP), Av. do Café, CEP 14040-903, Ribeirão Preto/SP, Brazil. e-mail: gclososki@usp.br (G.C.C.); gabrieldepaulabueno@hotmail.com (G.P.B.)

*Corresponding author. Tel.: +55-38-35321234; fax: +55-38-35321234; e-mail: sandro.barbosa@ufvjm.edu.br

1. List of Chemicals

Table 1. The list of chemicals (reagents and CAS number).

**Reagents**	**CAS number**
Acetylsalicylic acid	50-78-2
Methyl salicylate	119-36-8
Methanol	67-56-1
Sodium sulfate	7757-82-6
Dulbecco’s Modified Eagle Medium (DMEM) (Gibco)	was prepared in deionized water, buffered with sodium bicarbonate (Synth, Brazil) and supplemented with fetal bovine serum (FBS) (Vitrocell Embriolife).
Streptomycin	57-92-1
ampicillin	69-53-4
3-(4,5-dimethylthiazol-2-yl)-2,5-diphenyltetrazolium bromide (MTT)	298-93-1
Dimethyl sulfoxide	67-68-5
Mueller Hinton Broth Medium (MHB) (Kasvi)	was prepared in deionized water.
Dimethyl sulfate	99.5 77-78
Mouse Fibroblast L929 cells and Murine melanoma B16F10-Nex2 cells	were obtained from the collection of the Laboratory of Nanomaterials and Lanotoxicology, where they were stored at -80 ^{o}C

2. NMR spectra, interpretation of ¹H NMR Spectrum and characterization data of MS compound.

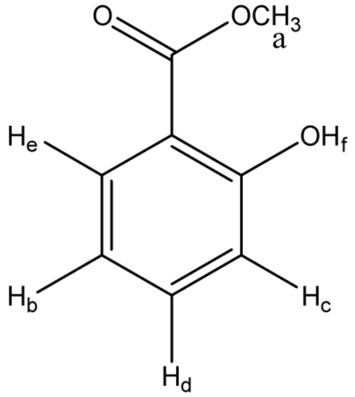

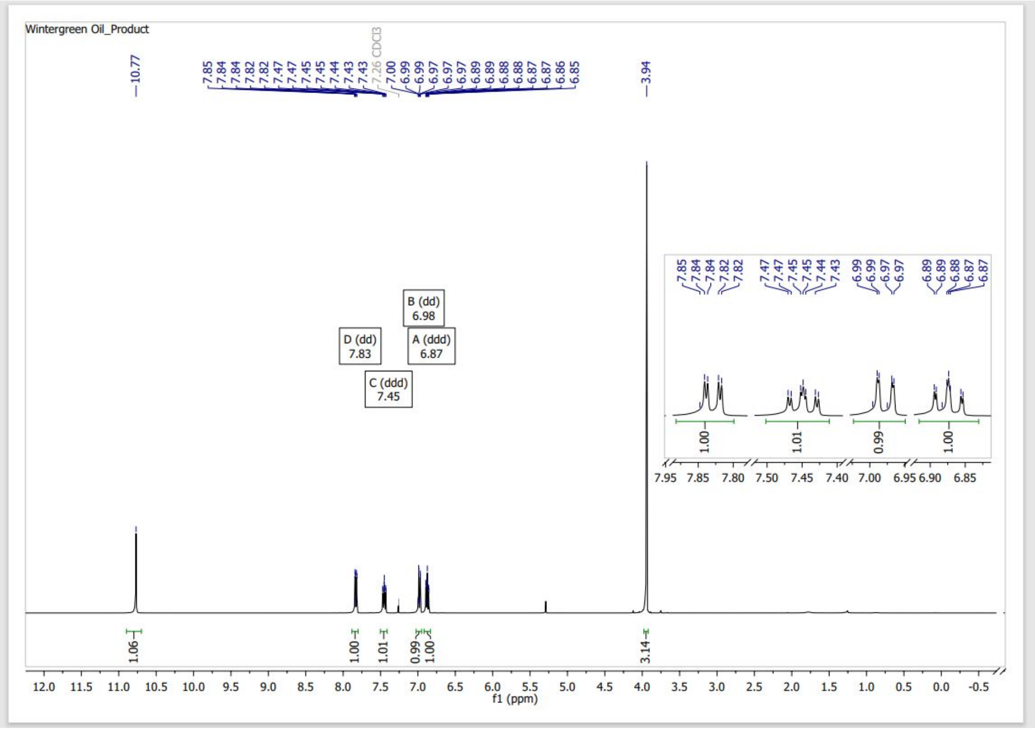

Figure S1. ¹H-NMR [400 MHz, $CDCl_3$, δ (ppm)] of MS: 3.94, s, 3H (CH_3); 6.88, ddd, 1H (H_b) (J_{be} = 8.0 Hz, J_{bd} = 7.1 Hz and J_{bc} = 1.1 Hz); 6.98, dd, 1H (H_c) (J_{cd} = 8.5 Hz, J_{cb} = 1.1 Hz); 7.45, ddd, 1H (H_d) (J_{dc} = 8.5 Hz, J_{db} = 7.1 Hz, J_{de} =1.7 Hz); 7.84, dd, 1H (H_e) (J_{eb} = 8.0 Hz, J_{ed} = 1.7 Hz) and 10.75, s, 1H (phenolic proton).

2.1. Interpretation of ¹H NMR Spectrum.

a – This hydrogen of methyl group (δ 3.94, s, 3 H) is unsplit and deshielded by the electronegative oxygen.

b This aromatic proton (δ 6.88, ddd, 1 H) is coupled through 3 bonds with proton e (J_{be} = 8.0 Hz) and proton d (J_{bd} = 7.1 Hz) and 4 bonds with proton c (J_{bc} = 1.1 Hz).

c This aromatic proton (δ 6.98, dd, 1 H) is coupled through 3 bonds with proton d (J_{cd} = 8.5 Hz) and 4 bonds with proton b (J_{cb} = 1.1 Hz).

d This aromatic proton (δ 7.45, ddd, 1 H) is coupled through 3 bonds with proton c (J_{dc} = 8.5 Hz) and proton b (J_{db} = 7.1 Hz) and 4 bonds with proton e (J_{de} =1.7 Hz).

e This aromatic proton (δ 7.84, dd, 1 H) is coupled through 3 bonds with proton b (J_{eb} = 8.0 Hz) and 4 bonds with proton d (J_{ed} = 1.7 Hz).

f This phenolic proton (δ 10.75, s, 1 H) is unsplit and is farther downfield than is typical for other phenols. This can be attributed to the hydrogen bonding that occurs with the carbonyl oxygen of the ester.

Note the much smaller coupling constants for the long range (4-bond) couplings. The coupling constants, splitting patterns and chemical shifts can be used to match each resonance to the corresponding proton of the compound. Proton a integrates as 3H, is unsplit and modestly deshielded by the neighboring oxygen methyl group. Identifying this as the methyl group of the ester is straightforward. Proton f integrates as 1H, is unsplit and far downfield, identifying this as the phenolic hydrogen. Identifying the aromatic protons is more involved. The hydroxyl group should shield protons b and c through electron donation to its ortho and para positions. The carbonyl should deshield protons d and e through electron withdrawal from its ortho and para positions. Proton b and proton e share a 3-bond coupling constant ($J_{be} = J_{eb}$ = 8.0 Hz) and are thus adjacent. Proton c and proton d share a 3-bond coupling constant ($J_{cd} = J_{dc}$ = 8.5 Hz) and are thus adjacent. Proton b and proton d share a 3-bond coupling constant (Jbd = Jdb = 7.1 Hz) and are thus adjacent. The smaller 4-bond coupling constants reinforce these identifications.

Proton b and proton c share a 4-bond coupling constant (Jbc = Jcb = 1.1 Hz) and thus are meta to each other. Proton d and proton e share a 4-bond coupling constant (Jde = Jed = 1.7 Hz) and thus are meta to each other.

2.2. NMR spectra, interpretation of ^{13}C NMR Spectrum and characterization data of MS compound.

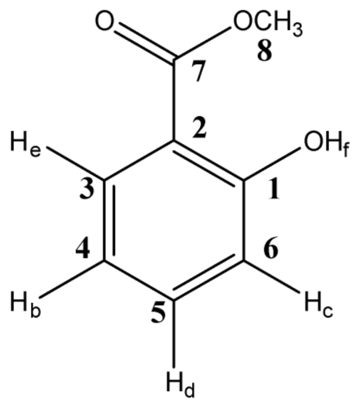

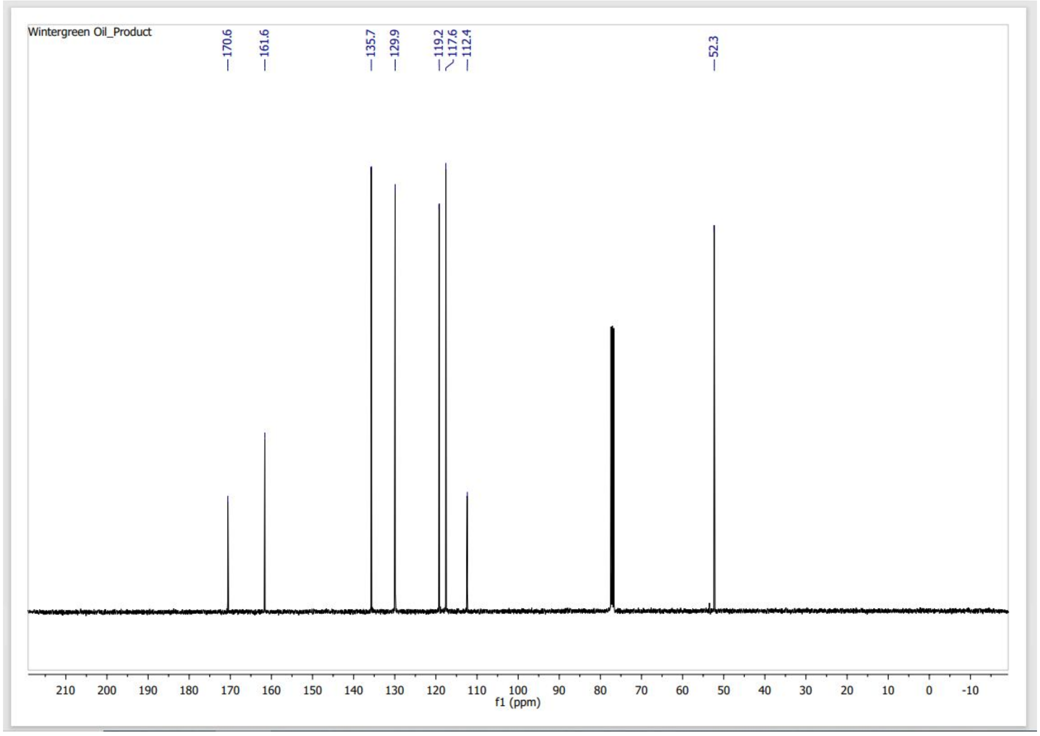

Figure S2. ^{13}C NMR [100 MHz, $CDCl_3$, δ (ppm)] of MS: 170.6 C=O or Ar quaternary, 161.6 C=O or Ar quaternary, 135.7 Ar CH, 129.9 Ar CH, 119.2 Ar CH, 117.6 Ar CH, 112.4 Ar quaternary and 52.3 CH_3.

2.3. Interpretation of ^{13}C NMR Spectrum.

Decoupled, eight peaks are observed, (ignoring the solvent peaks!). The methyl carbon is immediately recognizable due to its high field chemical shift. The carbonyl group is typically down field and could be either 170.6 or 161.6 ppm. Since quaternary carbons do not relax as quickly as tertiary, secondary and primary carbons then the peak intensities (and the chemical shifts!) indicate which peaks correspond to Ar CH and which to Ar quaternary carbons.

3. GC Chromatogram of MS compound.

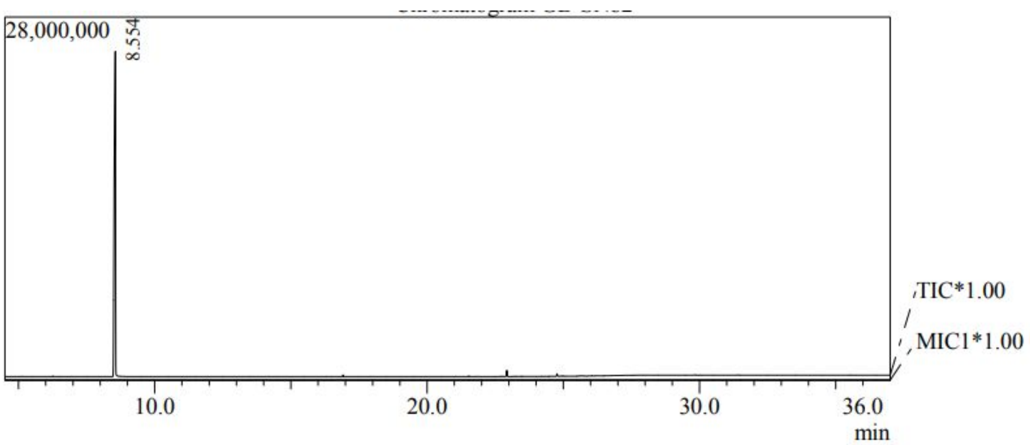

Peak Report TIC

Peak#	R.Time	Area	Area%	Name
1	8.554	83787596	100.00	Benzoic acid, 2-hydroxy-, methyl ester (
		83787596	100.00	

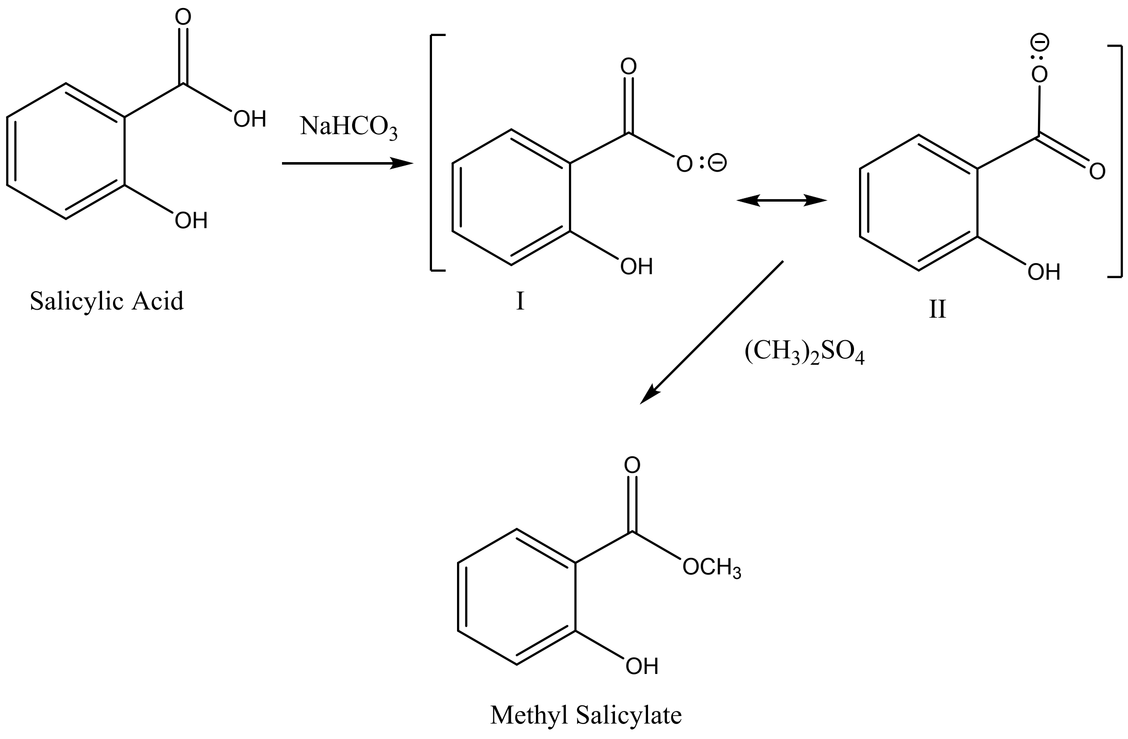

Scheme S1. Synthesis MS from SA using DMS as methylation agent.

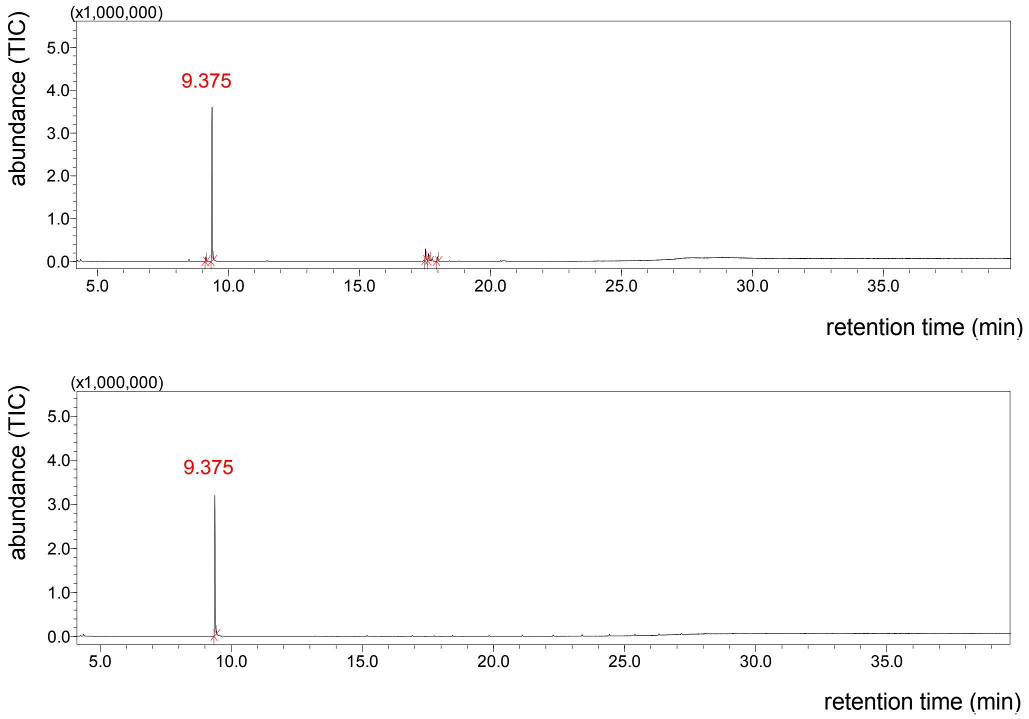

Figure S3. Cromatograms methylation process (upper) and purified product (inside).

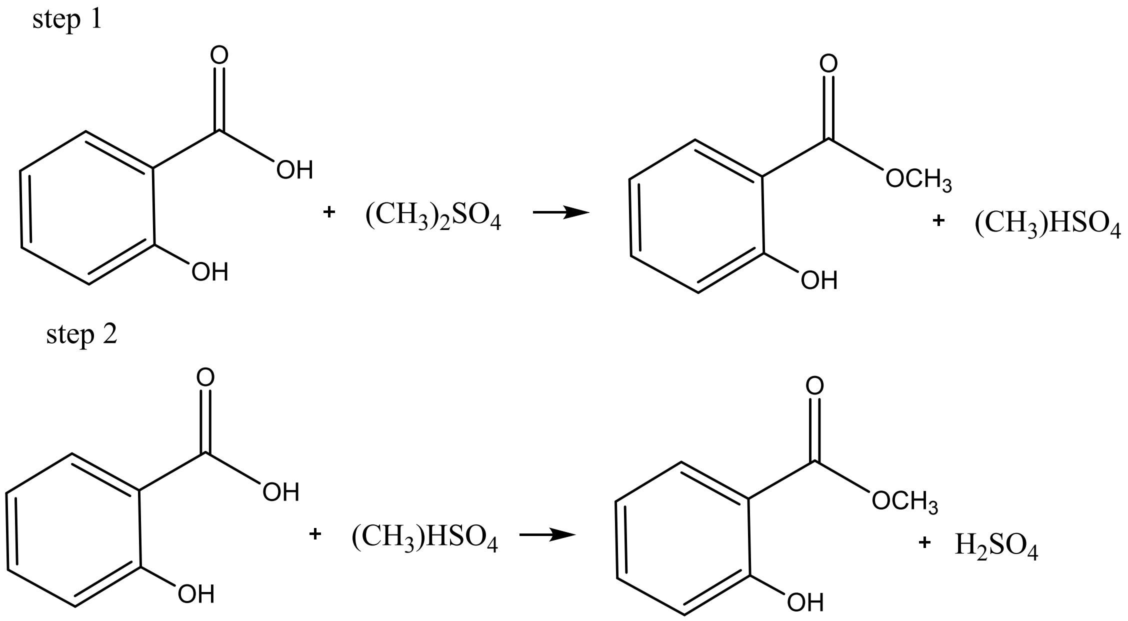

Scheme S2.

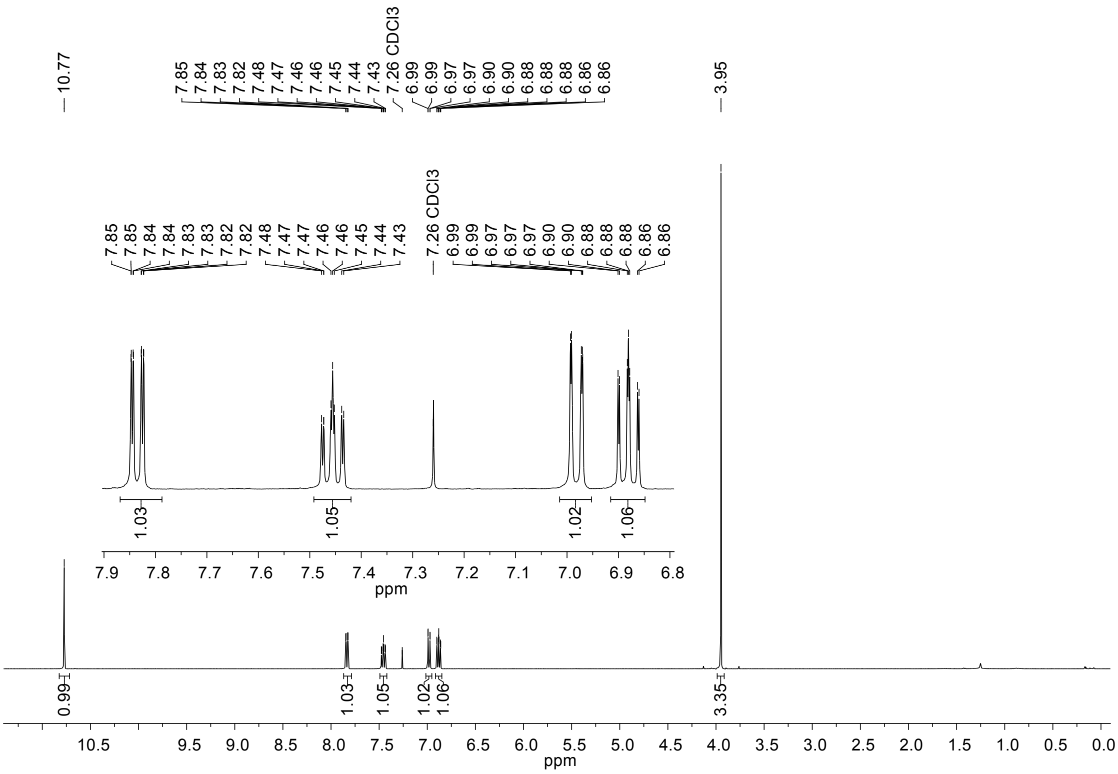

Figure S4. RMN ^1H (400 Hz, $CDCl_3$) of MS.

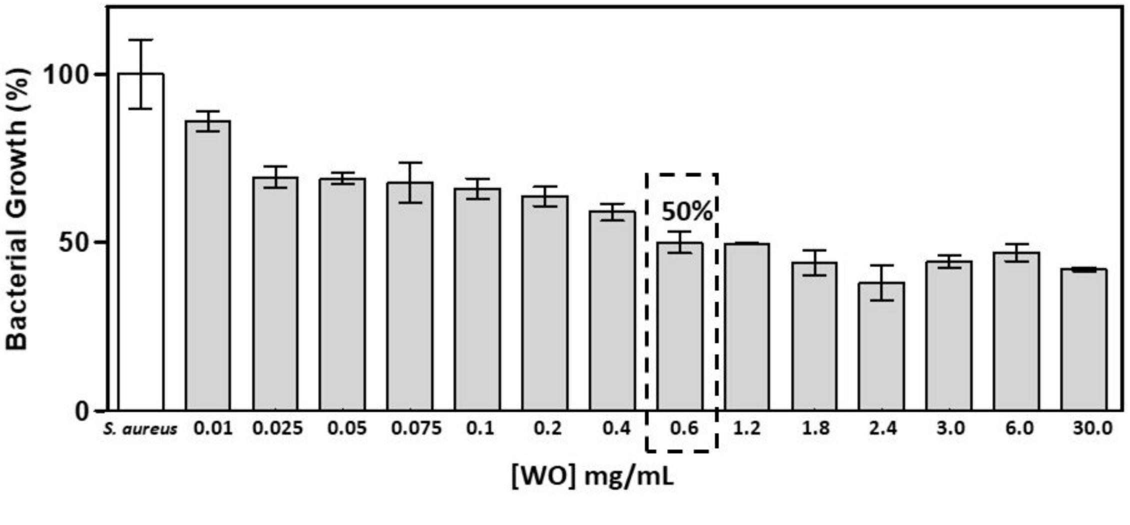

Figure. S5. Antimicrobial activity of WO against *S. aureus*.

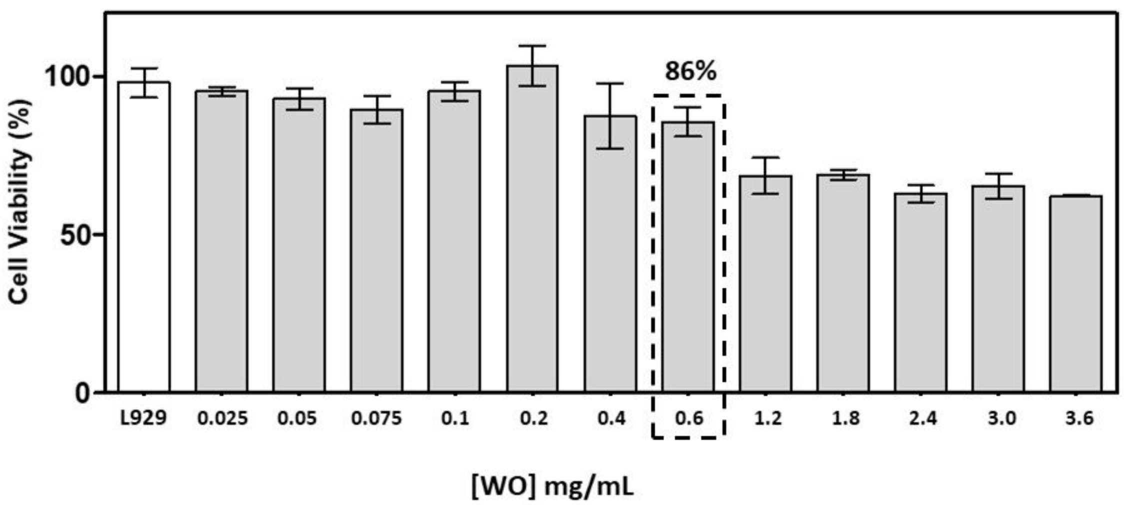

Figure S6. Cytotoxicity of WO in L929 cells.

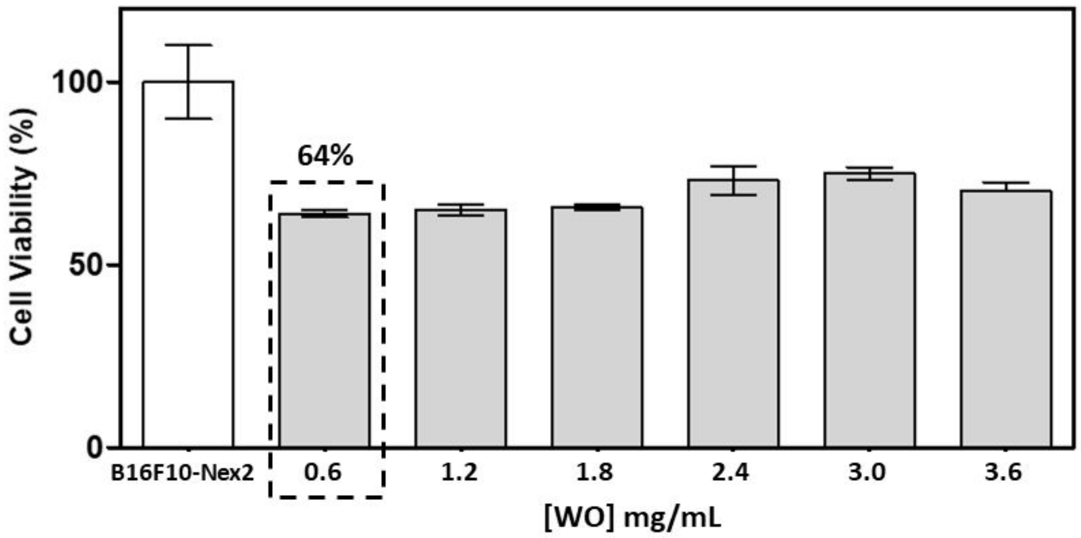

Figure S7. Cytotoxicity of WO in B16F10-Nex2 cells.
